# Spleen contributes to restraint stress induced changes in blood leukocytes distribution

**DOI:** 10.1038/s41598-017-06956-9

**Published:** 2017-07-26

**Authors:** Wei Jiang, Yu Li, Jin Sun, Liang Li, Jiang-wei Li, Chen Zhang, Chen Huang, Jun Yang, Guang-yao Kong, Zong-fang Li

**Affiliations:** 1 National & Local Joint Engineering Research Center of Biodiagnosis and Biotherapy, The second affiliated hospital of Xi’ an Jiaotong University, Xi’an, China; 2 Shaanxi Provincial Engineering Research Center of Biotherapy & Translational Medicine, The Second Affiliated Hospital of Xi’an Jiaotong University, Xi’an, China; 3 Shaanxi Provincial Clinical Research Center for Hepatic & Splenic Diseases, Xi’an, China; 4 Department of General Surgery, The second affiliated hospital of Xi’ an Jiaotong University, Xi’an, China; 5 Key Laboratory of Environment and Disease-Related Gene, Ministry of Education, Xi’an Jiaotong University, Xi’an, China; 6 Department of Pathology, The second affiliated hospital of Xi’ an Jiaotong University, Xi’an, China

## Abstract

Psychological stress has great impacts on the immune system, particularly the leukocytes distribution. Although the impacts of acute stress on blood leukocytes distribution are well studied, however, it remains unclear how chronic stress affects leukocytes distribution in peripheral circulation. Furthermore, there is no report about the role of spleen in the blood leukocytes distribution induced by stress. Here we show that spleen contributes to the alteration of restraint stress induced blood leukocytes distribution. Our data confirmed that restraint stress induced anxiety-like behavior in mice. Furthermore, we found that restraint stress decreased the CD4/CD8 ratio and elevated the percentages of natural killer cells, monocytes and polymorphonuclear myeloid-derived suppressor cell. We demonstrated that activation of hypothalamic-pituitary-adrenal axis (HPA) and sympathetic nervous system (SNS) contributes to restraint stress induced alteration of blood leukocyte distribution. Interestingly, we found that splenectomy could reverse the change of CD4/CD8 ratio induced by restraint stress. Together, our findings suggest that activation of HPA axis and SNS was responsible for the blood leukocyte subsets changes induced by restraint stress. Spleen, at least in part, contributed to the alteration in peripheral circulation induced by restraint stress.

## Introduction

Chronic stress is known to have many adverse effects on human health^1^. It is important to investigate the psychological and biological mechanisms by which chronic stress weaken health or exacerbate disease, which will enable the development of biobehavioral and pharmacological treatments to ameliorate the adverse effects of chronic stress. Previous studies demonstrate that psychosocial and emotional stress affect disease outcome through hindering or exacerbating immune response^2–4^. As peripheral circulation is essential for the maintenance of an effective immune defense network^5^, the numbers and proportions of leukocytes in the blood provide an important representation of the state of distribution of leukocytes in the body and of the state of activation of the immune system. Numerous studies have shown that short-term stress induces significant changes in absolute numbers and relative proportions of leukocytes in the blood^6–8^. However, most of the research investigated the leukocyte distribution induced by acute stress but not chronic stress. A more detailed understanding of blood leukocytes distribution and its potential mechanism induced by chronic stress is required.

Spleen is the largest secondary immune organs and plays a critical role in the disease development^9^, such as tumor^10–12^, myocardial infarction^13^, liver fibrosis^14^, and stroke^15^. Stress also induced significant changes in spleen leukocytes. Repeated social disruption increased percentages in splenic CD11b^+^ myeloid cells, granulocytes, CD11c^+^ dendritic cells while decreased NK cells^16–18^. Restraint stress decreased B cells and T cells in spleen^19^. However, there is no report about the role of spleen in the stress-induced changes of blood leukocyte distribution.

The purposes of this study were to investigate the effects of restraint stress on blood leukocyte subsets distribution and the role of spleen in stress-induced changes of blood leukocyte subsets. Here we showed that 21 cycles of restraint stress significantly changed the percentages of leukocyte subsets while 1, 7 cycles of restraint stress did not. In addition, blockade of the HPA axis activation or SNS activation could partially reverse restraint stress induced blood leukocyte redistribution. Moreover, splenectomy 14 days before restraint stress prevented the changes of CD4/CD8 ratio induced by restraint stress. Taken together, these data showed activation of HPA axis and SNS was responsible for the blood leukocyte subsets changes induced by restraint stress and splenectomy partially prevented the changes of leukocyte subsets induced by restraint stress.

## Results

### Restraint stress induced anxiety-like behavior

**Figure 1:**
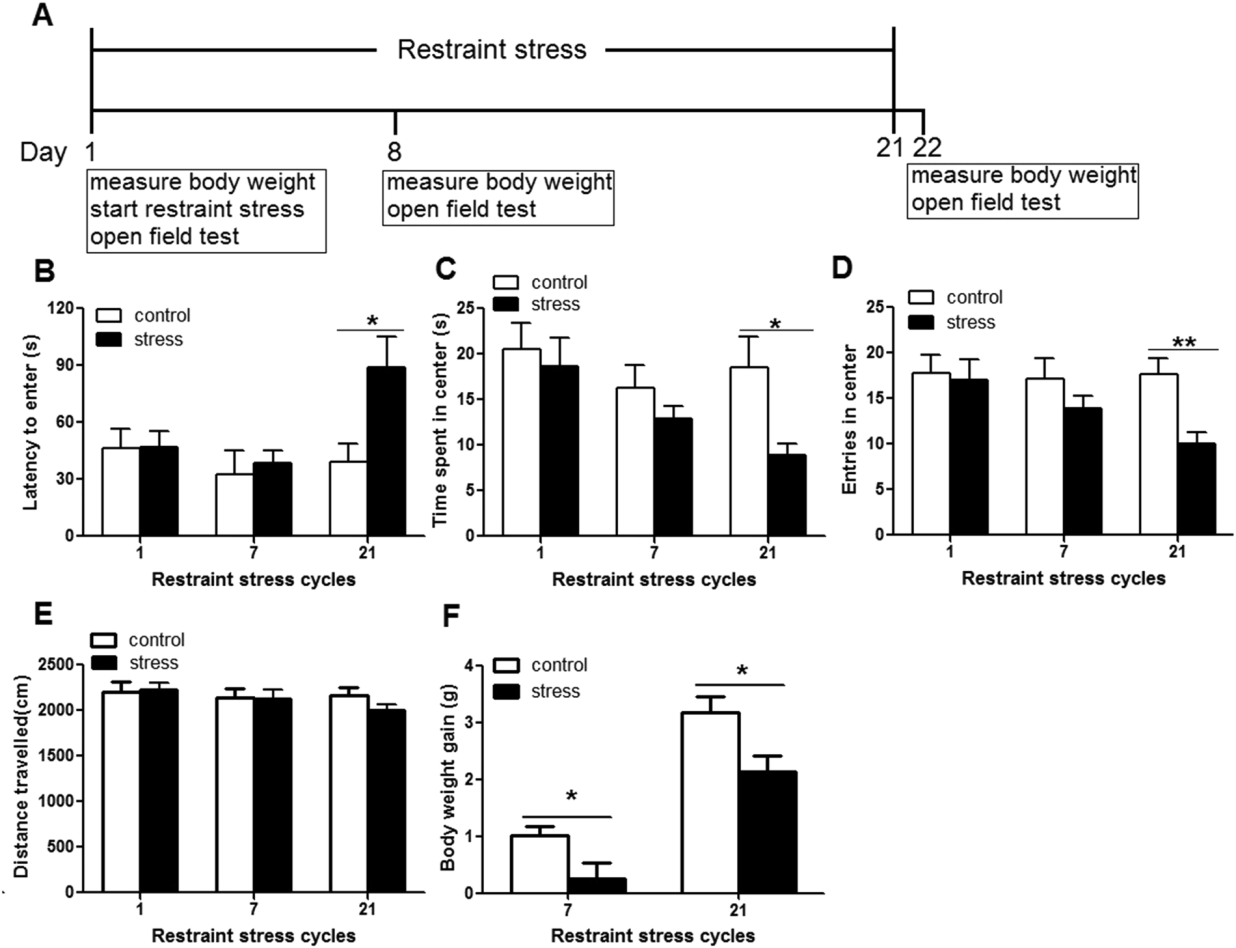
Restraint stress induced anxiety-like behavior in mice. Male C57BL/6 mice undergoing 1, 7 or 21 cycles of restraint stress (stress) or left undisturbed in home cage (control). (**A**) Experiment designs to test the impact of the restraint stress on mice. (**B**,**C** and **D**) Mice subjected to 21 cycles of restraint stress took longer to first enter the center of the open field (**B**), spent less time in the center of the open field (**C**), entered the center of the open field less often (**D**) than the controls. (**E**) There was no difference of the distance traveled in the open field between the restraint stressed mice and the control mice. (**F**) Body weight gain was reduced significantly after the mice undergoing 7 or 21 cycles of restraint stress. Data were shown as mean ± SEM (n = 10–15). *p < 0.05, **p < 0.01.

Mice were subjected to 2-hour restraint stress each day for 21 consecutive days (Fig. 1A). To study the impact of the restraint stress, we measured anxiety-like behavior by using open field test at different time point. We found that mice undergoing 1 cycle or 7 cycles of restraint stress took similar time to first enter the center of the open filed, spent similar time in the center of the open field and showed similar frequency to enter the center of the open filed compare with control mice (Fig. 1B,C and D), which suggest that short-term restraint stress did not affect anxiety-like behavior. However, mice undergoing 21 cycles of restraint stress displayed increased anxiety-like behavior in the open field test. Our data showed that mice subjected to 21 cycles of restraint stress took longer to first enter the center of the open field than the controls (control mice 30.88 ± 9.74 s, stress mice 88.98 ± 16.17 s; p = 0.025; Fig. 1B). Meanwhile, the restraint stressed mice spent less time in the center of the open field (control mice 18.56 ± 3.3 s, stress mice 8.95 ± 1.26 s; p = 0.018 Fig. 1C). Furthermore, restraint stressed mice entered the center of the open field less often than the controls (control mice 17.69 ± 5.52, stress mice 10.00 ± 4.99 s; p = 0.003; Fig. 1D). There was no difference of the distance traveled in the open field between the restraint stressed mice and the control mice (control mice 2167.63 ± 89.28 cm, stress mice 1996.29 ± 68.83 cm; p = 0.086; Fig. 1E), indicating that differences were not due to changes in locomotion or activity. Body weight was measured before starting stress, the day after the mice subjected to 7 cycles and 21 cycles of stress. Comparing to the control animals, body weight gain was reduced significantly after the mice undergoing 7cycles (control mice 1.01 ± 0.17 g, stress mice 0.18 ± 0.27 g; p = 0.031), and 21cycles (control mice 3.18 ± 0.28 g, stress mice 2.13 ± 0.28 g; p = 0.019) of restraint stress (Fig. 1F), which suggests that restraint stress attenuates body weight gain. Our data imply that only long-term restraint stress induces anxiety-like behavior in mice.

### Restraint stress changed blood leukocyte distribution

**Figure 2:**
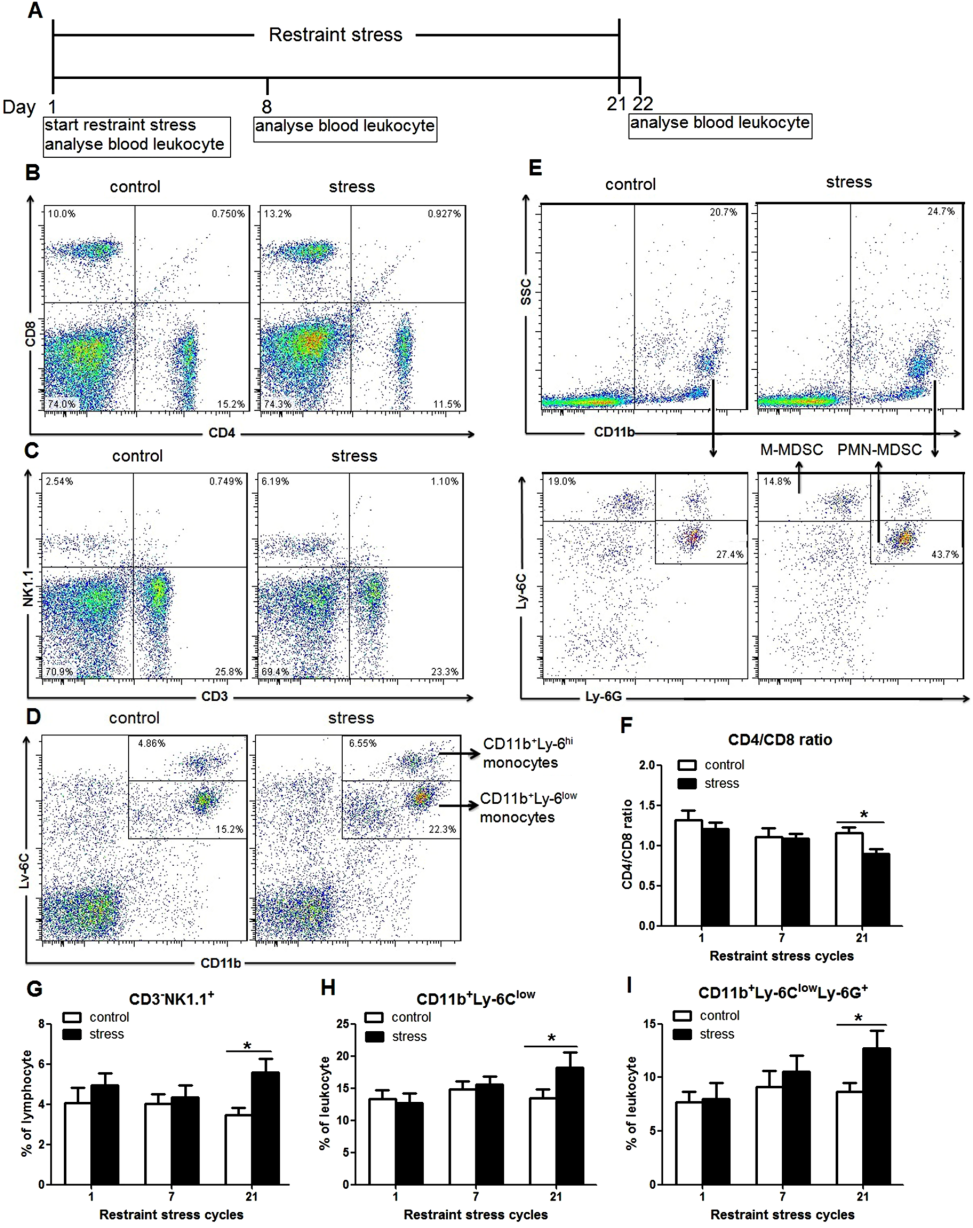
Leukocyte subsets of blood were changed after restraint stress. Blood was collected from the mice 1 h following the 1st cycle restraint stress, the day after undergoing 7 or 21 cycles of restraint stress. (**A**) Experiment designs to examine the changes of Leukocyte subsets of blood after restraint stress. (**B**,**C**,**D** and **E**) Representative dot plots of CD4^+^, CD8^+^ T lymphocytes (**B**), CD3^−^NK1.1^+^ NK cells (**C**), CD11b^+^Ly-6^low^ monocytes (**D**), and CD11b^+^Ly-6C^low^Ly-6G^+^ PMN-MDSCs (**E**) in the mice undergoing 21 cycles of restraint stress and controls. (**F**) Twenty-one cycles of restraint stress decreased CD4/CD8 ratio. (**G**,**H** and **I**) Twenty-one cycles of restraint stress elevated the percentage of CD3^−^NK1.1^+^ NK cells (**G**), CD11b^+^Ly-6^low^ monocytes (**H**) and CD11b^+^Ly-6C^low^Ly-6G^+^PMN-MDSCs (**I**). Data were shown as mean ± SEM (n = 8). *p < 0.05 vs. control.

To examine the effect of restraint stress on blood leukocytes distribution, we analyzed total leukocytes in blood by flow cytometric analysis (Fig. 2A). Our data indicated that 21 cycles of restraint stress did not significantly alter the percentages of CD3^+^CD4^+^ and CD3^+^CD8^+^ T lymphocytes, but decreased the CD4/CD8 ratio (control mice 1.15 ± 0.08, stress mice 0.89 ± 0.07; p = 0.036; Fig. 2B and F). 21 cycles of restraint stress also elevated the percentages of CD3^−^NK1.1^+^ NK cells (control mice 3.46% ± 0.35%, stress mice 5.60% ± 0.65%; p = 0.012; Fig. 2C and G), CD11b^+^Ly-6^low^ monocytes (control mice 13.43% ± 1.41%, stress mice 18.19% ± 2.46%; p = 0.046; Fig. 2D and H), CD11b^+^Ly-6C^low^Ly-6G^+^ polymorphonuclear MDSCs (PMN-MDSCs) (control mice 8.65% ± 0.86%, stress mice 12.70% ± 1.64%; p = 0.036; Fig. 2E and I). While, 21 cycles of restraint stress did not alter the percentages of CD3^−^CD19^+^ B lymphocytes, CD3^+^NK1.1^+^ NKT cells, CD11b^+^Ly-6^hi^ monocytes, CD11b^+^Ly-6G^+^ granulocytes, CD11b^+^Ly-6C^hi^Ly-6G^−^ monecytic MDSCs (M-MDSCs), and CD11c^+^MHCII^+^ DC cells (data not shown). Meanwhile, 1 cycle or 7 cycles of restraint stress did not change the percentage of leukocyte subsets in blood (Fig. 2). Thus, only long-term restraint stress affects blood leukocytes distribution.

### Restraint stress increased circulating norepinephrine and corticosterone

**Figure 3:**
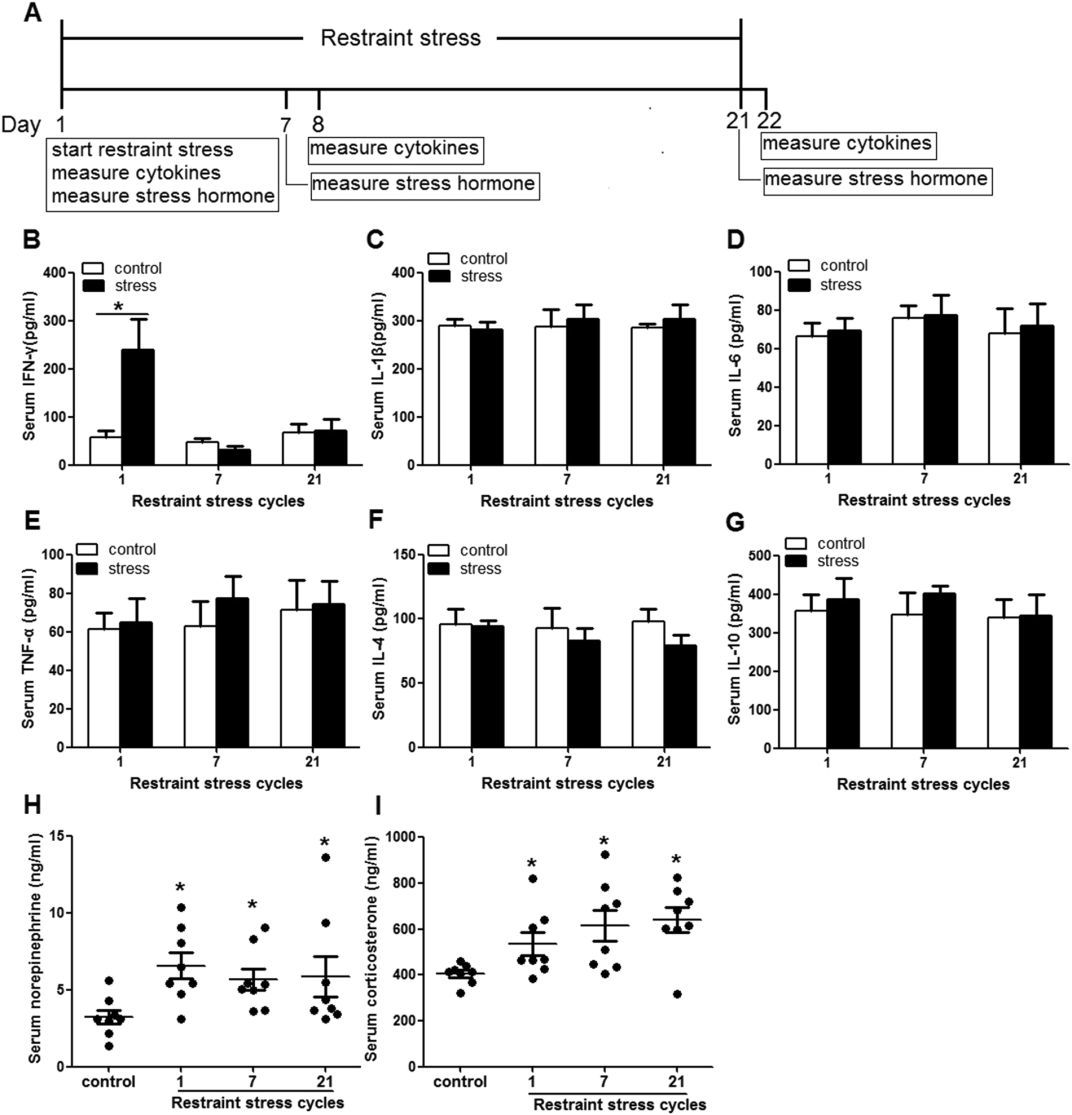
Restraint stress increased serum norepinephrine and corticosterone. Immediately following the 1, 7 or 21st cycles of restraint stress, blood was drawn from the retro-orbital plexus to determine norepinephrine and corticosterone. (**A**) One h after the mice subjected the first cycle stress, the day after mice subjected 7 or 21 cycles stress, blood was collected to determine cytokines. (**B**) The level of serum IFN-γ increased 1 h following the 1st cycle of restraint stress. (**C**,**D**,**E**,**F** and **G**) The level of other cytokines was not significantly different as compared to the controls. (**H** and **I**) The stressed mice displayed significantly elevated the concentration of (**H**) serum norepinephrine and (**I**) serum corticosterone as compared to the control animals. Data were shown as mean ± SEM (n = 5–8). *p < 0.05 vs. control.

Previous study indicates that chronic stress elevated level of plasma IL-6 and TNF-α the day after the last cycles of stress^20^. Therefore, we determined to assess the level of IL-1β, IL-6, TNF-α, IFN-γ, IL-4, and IL-10 in serum after undergoing restraint stress (Fig. 3A). Our results showed that only the level of serum IFN-γ increased 1 h following the 1st cycle of restraint stress (control 56.61 ± 15.21 pg/ml, 1 cycle of stress 239.54 ± 63.62 pg/ml; p = 0.028; Fig. 3B). The level of other cytokines was not significantly different as compared to the controls (Fig. 3C,D,E,F,G). Our data suggest that serum cytokines are not involved in the restraint stress induced anxiety-like behavior.

As norepinephrine and corticosterone were the physiologic parameters of stress^21^, norepinephrine and corticosterone were measured in restraint stressed mice and control animals. Immediately after the stressed mice subjected to 1, 7 or 21 cycles of restraint stress, approximately 1 ml of blood was drawn from the retro-orbital plexus of the stressed and control mice. The stressed mice after undergoing 1, 7 or 21 cycles of restraint stress displayed significantly elevated serum norepinephrine concentration as compared to the control animals (control 3.24 ± 0.45 ng/ml, 1 cycle of stress 6.95 ± 0.86 ng/ml, 7 cycles of stress 5.68 ± 0.70 ng/ml, 21 cycles of stress 5.85 ± 1.32 ng/ml; p < 0.05 vs. control; Fig. 3H). Similarly, restraint stress also elevated the level of serum corticosterone (control 404.94 ± 15.47 ng/ml, 1 cycle of stress 534.57 ± 51.39 ng/ml, 7 cycles of stress 613.23 ± 67.44 ng/ml, 21 cycles of stress 640.78 ± 54.53 ng/ml; p < 0.05 vs. control; Fig. 3I).These results demonstrate that restraint stress may induce anxiety-like behavior through elevating circulating norepinephrine and corticosterone.

### Activation of hypothalamic-pituitary-adrenal axis (HPA) and sympathetic nervous system (SNS) is involved in the altered leukocyte distributions in peripheral circulation

**Figure 4:**
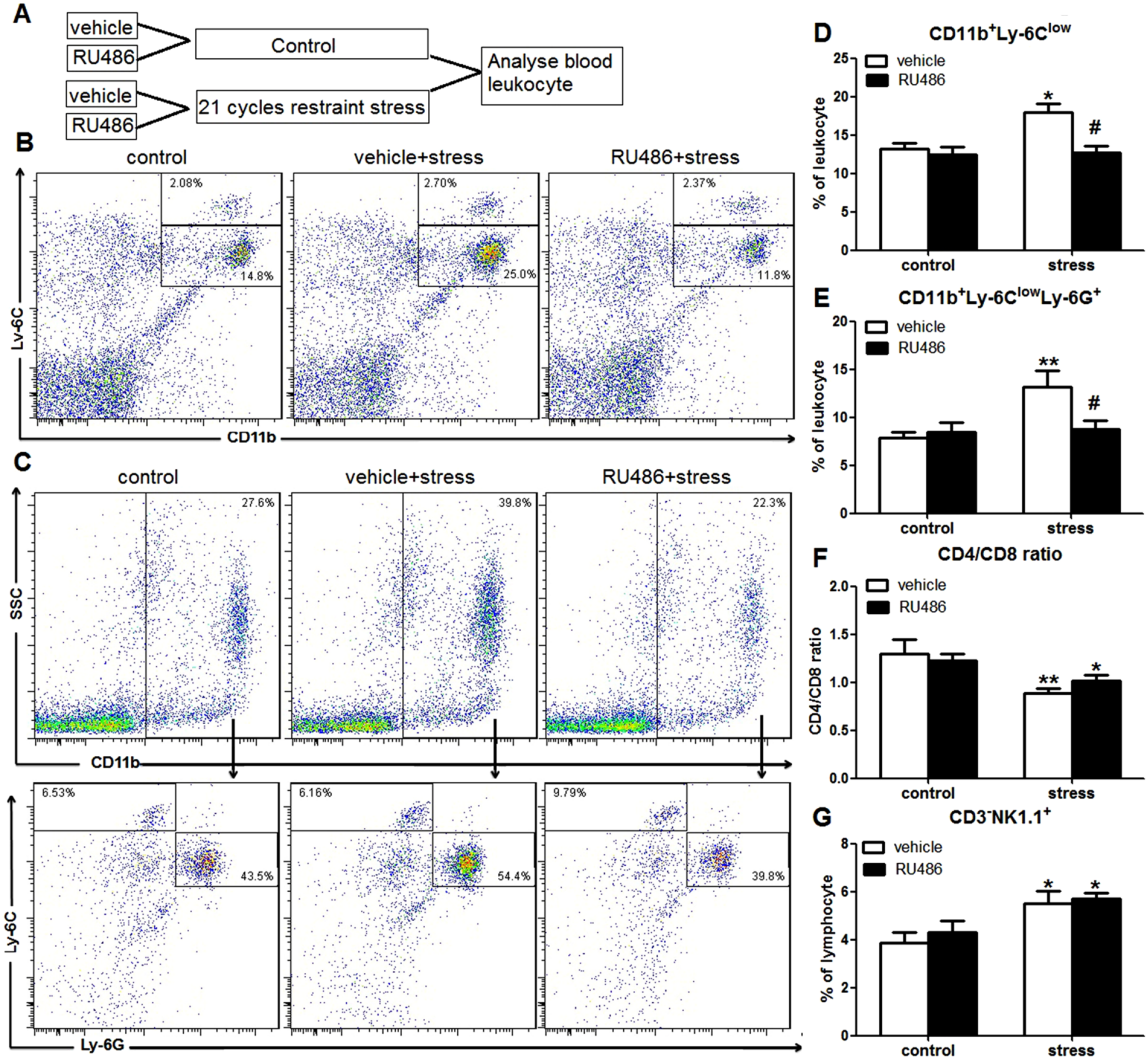
RU486 could attenuate the increase of the percentages of CD11b^+^Ly-6^low^ monocytes and CD11b^+^Ly-6C^low^Ly-6G^+^ PMN-MDSCs induced by restraint stress. (**A**) Mice were injected *sc* with vehicle or RU486 prior to restraint stress each day for 21 days. (**B** and **C**) Representative plots of CD11b^+^Ly-6^low^ monocytes (**B**) and CD11b^+^Ly-6C^low^Ly-6G^+^ PMN-MDSCs (**C**) in blood. (**D** and **E**) The increased percentages of CD11b^+^Ly-6^low^ monocytes (**D**) and CD11b^+^Ly-6C^low^Ly-6G^+^ PMN-MDSCs (**E**) were blocked by RU486. (**F** and **G**) RU486 could not attenuate the changes of the CD4/CD8 ratio (**F**) and the percentages of NK cells (**G**). Data were shown as mean ± SEM (n = 5). *p < 0.05, **p < 0.01 vs. control. ^#^p < 0.05 vs. vehicle.

Chronic restraint stress elevated levels of serum corticosterone immediately after the last cycle of stress. To determine if corticosterone antagonism would attenuate the changes of percentages of leukocyte subsets induced by chronic restraint stress, mice were injected *sc* with vehicle or RU486 prior to restraint stress each day for 21 days (Fig. 4A). The increased percentage of CD11b^+^Ly-6^low^ monocytes was blocked by RU486 (stress × RU486 interaction, F(1,18) = 5.232; p = 0.036; Fig. 4D). Similarly, the increased percentage of CD11b^+^Ly-6C^low^Ly-6G^+^ PMN-MDSCs was also blocked by RU486 (stress × RU486 interaction, F(1,18) = 5.036; p = 0.039; Fig. 4E). However, RU486 could not attenuate the changes of the CD4/CD8 ratio (stress × RU486 interaction, F(1,18) = 1.088; p = 0.312; Fig. 4F), and the percentages of NK cells (stress × RU486 interaction, F(1,18) = 0.084; p = 0.774; Fig. 4G).

**Figure 5:**
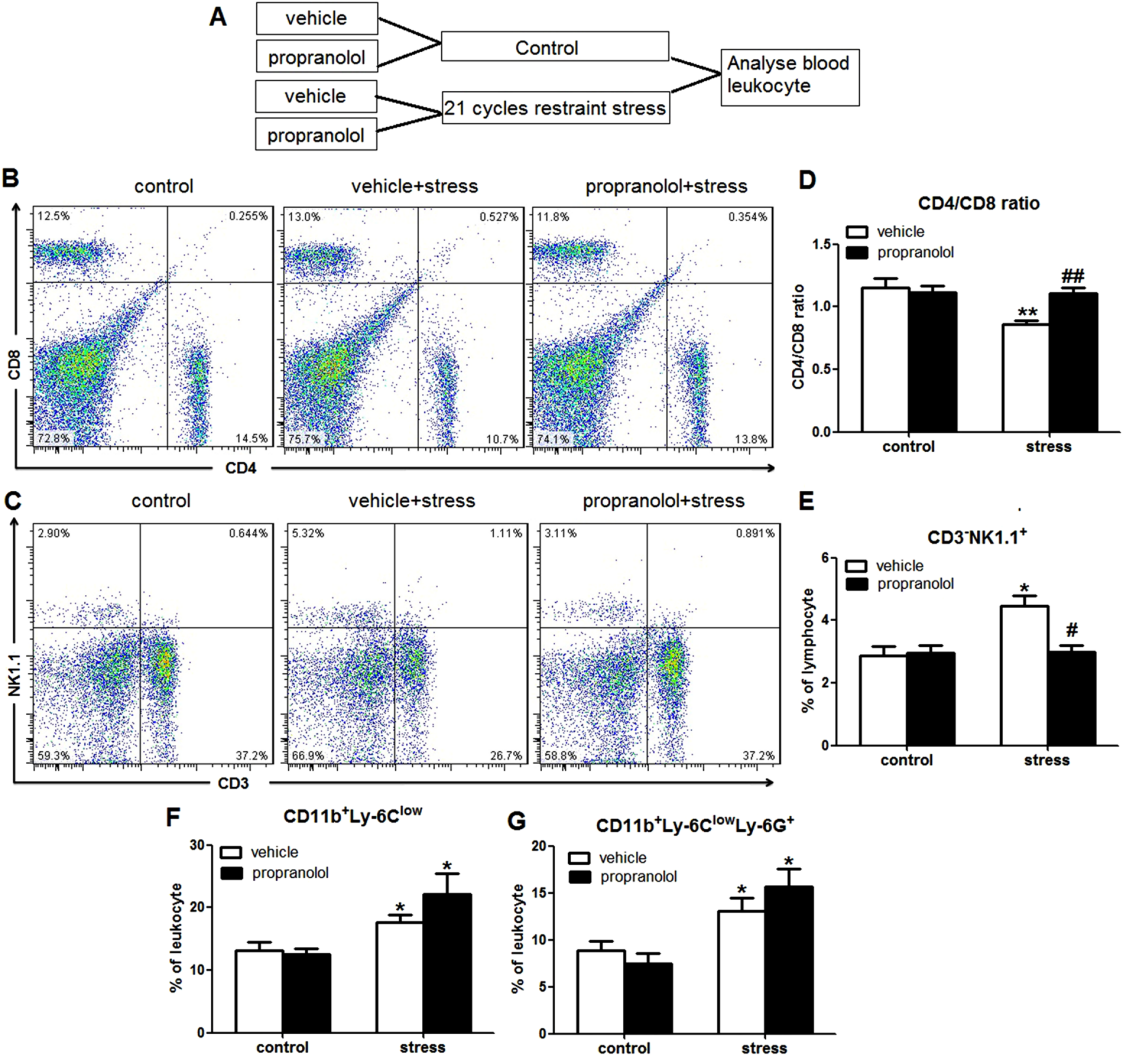
Propranolol could attenuate the changes of the CD4/CD8 ratio and the percentages of NK cells induced by restraint stress. (**A**) Mice were injected *sc* with vehicle or proraonlol prior to restraint stress each day for 21 days. (**B** and **C**) Representative plots of CD4^+^, CD8^+^ T lymphocytes (**B**) and NK cells (**C**) in blood. (**D** and **E**) The changes of the CD4/CD8 ratio (**D**) and the percentages of NK cells (**E**) were blocked by proraonlol. (**F** and **G**) Proraonlol could not attenuate the changes of the percentages of CD11b^+^Ly-6^low^ monocytes (**F**) and CD11b^+^Ly-6C^low^Ly-6G^+^ PMN-MDSCs (**G**). Data were shown as mean ± SEM (n = 6). *p < 0.05, **p < 0.01 vs. control. ^#^p < 0.05, ^##^p < 0.01 vs. vehicle.

In addition to increased serum corticosterone, chronic restraint stress also increased the levels of serum norepinephrine. Recent studies indicate that stress-induced activation of sympathetic nervous system (SNS) mediates effects on immune through beta(β)-adrenergic receptor^18, 22^. To determine if beta(β)-adrenergic receptor antagonism would attenuate the changes of percentages of leukocyte subsets induced by chronic restraint stress, mice were injected *sc* with vehicle or propranolol prior to restraint stress each day for 21 days (Fig. 5A). The changes of the CD4/CD8 ratio (stress × propranolol interaction, F(1,22) = 9.027; p = 0.007; Fig. 5D) and the percentages of NK cells (stress × propranolol interaction, F(1,22) = 7.602; p = 0.012; Fig. 5E) were also blocked by propranolol. But, propranolol could not reverse the changes of the percentages of CD11b^+^Ly-6^low^ monocytes (stress × propranolol interaction, F (1,22) = 1.230; p = 0.280; Fig. 5F) and CD11b^+^Ly-6C^low^Ly-6G^+^ PMN-MDSCs (stress × propranolol interaction, F(1,22) = 2.061; p = 0.167; Fig. 5G). These data indicated that RU486 or propranolol treatment could partially reverse the alteration in peripheral circulation in stressed mice, which supports that activation of hypothalamic-pituitary-adrenal axis (HPA) and sympathetic nervous system (SNS) is involved in the altered leukocyte distributions in peripheral circulation.

### Splenectomy reversed the changed CD4/CD8 ratio caused by restraint stress

**Figure 6:**
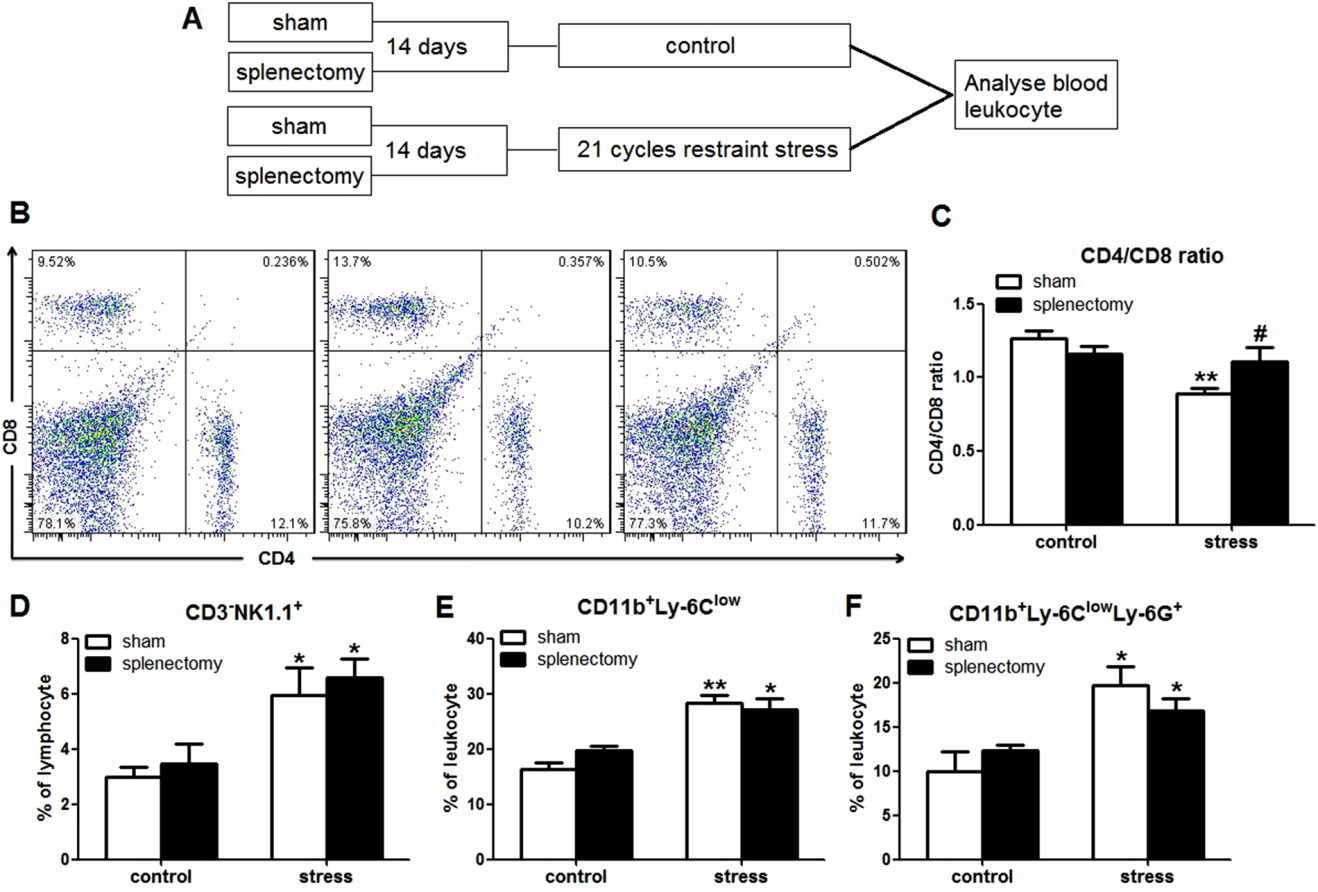
Splenectomy before restraint stress reversed the CD4/CD8 ratio changing following 21 cycles of stress. (**A**) Mice were splenectomized 14 days before restraint stress and then exposed to 21 cycles of restraint stress. (**B**) Representative plots of CD4^+^, CD8^+^ T lymphocytes in blood. (**C**) Splenectomy before restraint stress could attenuate the changes of CD4/CD8 ratio. (**D**,**E** and **F**) Splenectomy could not block the changes of NK cells (**D**), CD11b^+^Ly-6^low^ monocytes (**E**), CD11b^+^Ly-6C^low^Ly-6G^+^ PMN-MDSCs (**F**) induced by restraint stress. Data were shown as mean ± SEM (n = 5–6). *p < 0.05, **p < 0.01 vs. control. ^#^p < 0.05 vs. sham.

Our previous study showed that 21 cycles of restraint stress changed the CD4/CD8 ratio, reduced the percentages of CD11b^+^Ly-6^low^ monocytes and CD11b^+^Ly-6C^low^Ly-6G^+^ PMN-MDSCs in spleen (Yu Li *et al*., in the submission).To determine if splenectomy before restraint stress could affect the changes of leukocyte subsets distribution, mice were splenectomized 14 days before restraint stress and then exposed to 21 cycles of restraint stress (Fig. 6A). Splenectomy before restraint stress attenuated the changes of CD4/CD8 ratio (stress × splenectomy interaction, F (1,19) = 6.635; p = 0.020; Fig. 6C). However, our results showed that splenectomy could not block the changes of NK cells (stress × splenectomy interaction, F (1,19) = 0.016; p = 0.902), CD11b^+^Ly-6^low^ monocytes (stress × splenectomy interaction, F (1,19) = 2.241; p = 0.153),CD11b^+^Ly-6C^low^Ly-6G^+^ PMN-MDSCs (stress × splenectomy interaction, F(1,19) = 2.438; p = 0.137) induced by restraint stress (Fig. 6D,E,F). Our findings suggest that spleen, at least in part, is involved in the restraint stress induced changes in blood leukocytes distribution.

## Discussion

These results of our study demonstrate a novel role of spleen in the restraint stress-induced blood leukocyte subsets changes. First, we have shown that restraint stress induced HPA axis and SNS activation, a delay in weight gain, and an increase in anxiety-like behavior. Second, restraint stress did not alter the serum cytokine but one cycle restraint stress increased the serum IFN-γ. Third, corticosterone antagonism blocked the 21 cycles of restraint stress induced the increase of the percentages of CD11b^+^Ly-6^low^ monocytes and CD11b^+^Ly-6C^low^Ly-6G^+^ PMN-MDSCs. Fourth, beta(β)-adrenergic receptor antagonism attenuates the changes of the CD4/CD8 ratio, and the percentages of NK cells induced by 21 cycles of restraint stress. Finally, splenectomy before restraint stress reversed the changed CD4/CD8 ratio following 21 cycles of restraint stress.

Our data shown the restraint stress induced a delay in weight gain, an increase in anxiety-like behavior, indicating our stress model was effective^3, 23, 24^.Our results showed chronic restraint stress did not alter serum cytokine but the level of serum IFN-γ increased 1 h following the 1st cycle of restraint stress. However, previous studies reported chronic stress increased the plasma level of IL-6and TNF-α^24, 25^, while another report showed that restraint stress caused a significant decrease in the concentrations of circulating IFN-γ, IL-12(p40), IL-12(p70), IL-4, and IL-5^26^. Recently, *Smith et al*. reported chronic restraint stress and chronic variable stress caused different effect on immune^27^. Therefore, we propose that the differences in the level of serum cytokines may be caused by different animal model and the timing of sampling.

Previous study shown acute stress increased an early increase of leukocyte followed by a decrease, and began to increase and return to pre-stress baseline after the stressor was terminated^28, 29^, which is consistent with our results that 1 or 7 cycles of restraint stress did not change the percentage of leukocyte subsets in blood. Stress activates HPA axis and SNS to release corticosterone and catecholamines (adrenaline and norepinephrine). Previous researches shown T and B cells, neutrophils, monocytes and macrophages express corticosterone receptor while T and B cells, NK cells, monocytes and macrophages express adrenergic receptors^30^. The adrenergic receptors can be divided into two subgroups, the α- and β- adrenergic receptors, and the β- adrenergic receptor plays the most important role in the immune system^31^. Numerous studies have shown that acute stress induces alters in absolute numbers or/and relative proportions of blood leukocytes by stress hormones^28, 32–35^. Our results indicated that RU486 could block the increase of the percentages of CD11b^+^Ly-6^low^ monocytes and CD11b^+^Ly-6C^low^Ly-6G^+^ PMN-MDSCs while propranolol could block the change of the CD4/CD8 ratio, and the increase of NK cells induced by 21 cycles of restraint stress. In other words, chronic restraint stress induced alteration of blood leukocytes by activating HPA axis and SNS.

The splenectomy study provides first evidence that spleen plays an important role in the change of CD4/CD8 ratio induced by chronic restraint stress. Splenectomy could prevent the change of CD4/CD8 ratio induced by chronic restraint stress resembles the studies of liver fibrosis that splenectomy reduced Th2 lymphocytes^36^. Splenectomy before stress could not block the changes of CD11b^+^Ly-6^low^ monocytes and CD11b^+^Ly-6C^low^Ly-6G^+^ PMN-MDSCs. These results are consistent with *Mckin et al*. reported that splenectomy did not prevent increased monocytes in circulation following repeated social defeat stress^37^. Bone marrow may be to the initial production of accumulation of CD11b^+^Ly-6^low^ monocytes and CD11b^+^Ly-6C^low^Ly-6G^+^ PMN-MDSCs because stress increased production of myeloid cell in bone marrow^38^.

In summary, our findings provide suggestive evidence that activation of HPA axis and SNS is responsible for the blood leukocyte subsets changes induced by chronic restraint stress. Spleen, at least in part, contributes to the changes of blood leukocyte subsets induced by chronic restraint stress.

## Materials and Methods

### Mice

Male C57BL/6 mice (6–8 weeks old) were obtained from Experimental Animal Center of Xi’an Jiaotong University College of Medicine and allowed to acclimate the surroundings for 14 days before the experimental procedure started. Mice were housed 5 animals per cage under specific pathogen-free condition and had free access to tap water and standard mouse diet. The study was approved by Ethics Committee of Xi’an Jiaotong University College of Medicine according to the principles outlined in the Declaration of Helsinki.

### Restraint stress

Mice were subjected to restraint stress similar to the previously reported^39^. In brief, mice were placed in ventilated 50-ml conical tubes for 2 h each day between 1700 and 2100 h Beijing for 21 consecutive days. Both the stress and control mice were not available for food and water during the stress period. Weight was measured before starting stress, the day after the mice subjected 7 cycles, 21 cycles stress.

### Anxiety-like behavior

As previously described^40, 41^, open field test was applied to measured anxiety-like behavior. Mice were placed individually into the corner of open field chamber (45 × 45 × 45 cm) under a bright light (500 lux) for 5 min immediately after the mice subjected the first cycle stress, the day after mice subjected 7 and 21 cycles stress. Computer imaging video tracking system (SMART, Panlab SL, Barcelona, Spain) was applied to record the track. The dependent variables latency to entering the center, time spent in center, times of entering center were applied to measure the anxiety-like behavior. The chamber was cleaned with 75% alcohol between subjects.

### Serum cytokine measurement

1 h after the mice subjected the first cycle stress, the day after mice subjected 7 and 21 cycles stress, approximately 1.00 ml of blood was drawn from the retro-orbital plexus and serum stored at −80 °C for subsequent. Serum cytokine (IL-1β, IL-6, TNF-α, IFN-γ, IL-4, IL-10) were measured with ELISA kit (Westang, Shanghai, China) following the manufacturer’s instructions.

### Serum norepinephrine and cortcosterone measurement

Immediately after the mice subjected 1, 7, and 21 cycles of restraint stress, approximately 1.00 ml of blood was drawn from the retro-orbital plexus. The blood samples were allowed to clot at room temperature for 1 h, afterward, centrifuged at 5000 rpm for 10 min and serum stored at −80 °C for subsequent. Serum norepinephrine and cortcosterone were measured with mouse norepinephrine ELISA kit (Westang, Shanghai, China) and corticosterone Enzyme Immunoassay kit (Arbor Assays, Michigan, USA) following the manufacturer’s instructions, respectively.

### Flow cytometric analysis

Blood were collected in ethylenediaminetetraacetic acid (EDTA) coated tube. Red blood cells were lysed by adding 5 ml ammonium-chloride-potassium (ACK) lysis buffer (0.16 M NH_4_Cl, 10 mM KHCO_3_, 0.13 mM EDTA, PH 7.2) for 5 min in ice, followed by twice wash with phosphate buffered saline (PBS). Single cell suspensions (1 × 10^6^ cells per sample) were incubated with appropriate antibodies for 45 min at 4 °C. Afterward, cells were washed twice and detected by FACSCanto II flow cytometer (BD Biosciences, USA). Data were analysed using Flow Jo (Tree Star, Ashland, OR, USA). The following antibodies were purchased from BioLegend (San Diego, California, USA): FITC-labeled anti-CD3 (clone 17A2), PE-labeled anti-CD4 (clone GK1.5), APC-labeled anti-CD8a (clone 53–6.7), FITC-labeled anti-CD11c (clone N418), APC-labeled anti-CD11 (clone M1/70), PE-labeled anti-Ly6C (clone HK1.4), Brilliant Violet 421^TM^-labeled anti-CD19 (clone 6D5). While the following antibodies were purchased from eBioscience (San Diego, California, USA): PE-labeled anti-MHC II (clone M5/114.15.2), FITC-labeled anti-Ly6G (clone RB6-8C5), PerCp-Cyanine5.5 anti-NK1.1 (clone PK136).

### Pharmacological treatments

Mice were injected subcutaneously (*sc*) with either 100 ul sterile H_2_O, 100 ul olive oil, 25 mg/Kg corticosterone antagonism RU486 (MedChem Express, Princeton, NJ, USA), or 15 mg/Kg beta(β)-adrenergic receptor antagonism propranolol hydrochloride (Abcam, England, UK). For RU486, mice were injected 2 h before beginning stress each day. For propranolol hydrochloride, mice were injected 30 min before the beginning of stress each day.

### Splenectomy

Splenectomy was performed 14 days before the beginning of stress. Mice were anesthetized with chloral hydrate (100 ug/g body weight) and the skin in the left abdomen was shaved. A splenectomy was performed through a small incision in the abdomen. The associated vessels and nerves were ligatured. Afterward, the incision was sutured. Sham surgery was performed only made an incision in the abdomen without removal of the spleen.

### Statistical analysis

Data of obtained from open field test, body weight, stress hormone and cytokine ELISA’s, and flow cytometric procedures were analyzed by two-tailed non-parametric test (Mann-Whitney U-test); to determine the effects of stress and pharmacological treatments or splenectomy, data were analyzed by a two-way ANOVA (SPSS 13.0, Chicago, IL, USA). In all comparisons, differences were considered significant at p < 0.05. Data were shown as mean ± standard error of the mean (SEM).

### Data availability statement

All data generated during and analysed during the current study are available from the corresponding author on reasonable request.
